# Myostatin Changes in Females with UI after Magnetic Stimulation: A Quasi-Experimental Study

**DOI:** 10.3390/medicina60091399

**Published:** 2024-08-26

**Authors:** Maurizio Filippini, Simona Bugli, Nicoletta Biordi, Fausto Muccioli, Valentina Reggini, Milena Benedettini, Serena Migliore, Laura Pieri, Alessandra Comito, Beatrice Marina Pennati, Irene Fusco, Pablo Gonzalez Isaza, Antonio Posada Dominguez, Tiziano Zingoni, Miriam Farinelli

**Affiliations:** 1 Department of Obstetrics and Gynaecology, Hospital State of Republic of San Marino, 47893 San Marino, San Marino; maurizio.filippini@iss.sm (M.F.); simona.bugli@iss.sm (S.B.); adelitasm@yahoo.it (N.B.); serena.bartoli.migliore@gmail.com (S.M.); miriam.farinelli@iss.sm (M.F.); 2 Department of Transfusion Medicine and Clinical Pathology, Hospital State of Republic of San Marino, 47893 San Marino, San Marino; fausto.muccioli@iss.smn (F.M.); valentinareggini@yahoo.it (V.R.); millibsm@libero.it (M.B.); 3 El. En. Group, 50041 Florence, Italy; l.pieri@deka.it (L.P.); a.comito@elen.it (A.C.); b.pennati@deka.it (B.M.P.); t.zingoni@elen.it (T.Z.); 4 Division of Urogynecology and Pelvic Reconstructive Surgery, Department of Obstetrics and Gynecology, San Jorge University Hospital, Pereira 660002, Colombia; pagonza@hotmail.com; 5 Colsposcopy Unit, Department Obstetrics and Gynecology, Centro Hospitalario La Concepcion, Saltillo 25230, Mexico; antonioposada2000@yahoo.com

**Keywords:** myostatin, magnetic stimulation, stress urinary incontinence, pelvic floor, genital hiatus, ultrasound, quality of life

## Abstract

*Background and Objectives*: Urinary incontinence (UI) is the involuntary loss of urine caused by a weakness in the pelvic floor muscles (PFMs) that affects urethral closure. Myostatin, which prevents the growth of muscles, is a protein expressed by human skeletal muscle cells. Indeed, it has been observed that myostatin concentration rises during skeletal muscle inactivity and that suppressing serum myostatin promotes muscle growth and strength. Furthermore, therapeutic interventions that reduce myostatin signalling may lessen the effects of aging on skeletal muscle mass and function. For this reason, the aim of the study was to assess if flat magnetic stimulation technology affects serum myostatin levels, as myostatin can block cell proliferation at the urethral sphincter level. *Materials and Methods*: A total of 19 women, 75% presenting stress urinary incontinence (SUI) and 25% urgency urinary incontinence (UUI), were enrolled. A non-invasive electromagnetic therapeutic system designed for deep pelvic floor area stimulation was used for eight sessions. *Results*: The ELISA (enzyme linked immunosorbent assay) test indicated that the myostatin levels in blood sera had significantly decreased. Patients’ ultrasound measurements showed a significant genital hiatus length reduction at rest and in a stress condition. The Pelvic Floor Bother Questionnaire consistently revealed a decrease in mean scores when comparing the pre- and post-treatment data. *Conclusions*: Effective flat magnetic stimulation reduces myostatin concentration and genital hiatus length, minimizing the severity of urinary incontinence. The results of the study show that without causing any discomfort or unfavourable side effects, the treatment plan significantly improved the PFM tone and strength in patients with UI.

## 1. Introduction

Women’s quality of life (QoL) is impacted by urinary incontinence (UI), a prevalent disorder that can occur at any stage in life [1]. Some UI risk factors are age, pregnancy and childbirth (in multiparous women), pelvic floor injury during vaginal delivery, pelvic surgery, menopause (due to decreased oestrogen secretion), hysterectomy, increased body weight, and urinary tract infections [2].

The urinary incontinence caused by abdominal pressure is referred to as stress UI (SUI), followed by urgency UI (UUI), mixed UI (MUI), and overflow UI (also known as involuntary urine leakage caused by an overfilled bladder). Specifically, SUI [3] is the cause of around 50% of UI cases in female perimenopausal and postmenopausal populations and it can have a significant negative impact on a person’s physical, psychological, and social welfare. UUI refers to a complaint of involuntary leaking that is accompanied or preceded by urgency, while MUI constitutes a combination of SUI and UUI with the potential to mimic both pathologies. MUI is particularly frequently observed in women over 65, affecting more than 37% of older female patients [4].

Many approaches to treat UI have been studied: surgical procedures (such as urethral bulking agents), pharmacological treatments (such as anticholinergic medications), conservative therapies (such as pessaries, biofeedback, peripheral nerve stimulation, and electrical stimulation of the pelvic floor), physical therapies (Kegel exercises, vaginal weight training), and behavioural therapies (such as timed voiding and limited fluid intake) [5].

Treatment choices, whether conservative or surgical, are primarily based on the kind and severity of UI as well as co-occurring conditions. Patient’s age, overall health, and in particular dynamic and functional radiological imaging modalities can be utilised to determine the optimal course of treatment for a given patient [6]. Nonetheless, before considering surgery, all conservative measures should be attempted first [2]. Surgical therapies provide optimal results, but are invasive and may have risks and complications; therefore, more than 60% of patients would prefer not to undergo surgery and about 15% of patients need other procedures owing to recurrences. Indeed, conservative treatments continue to be the preferred way of treating SUI [7], contracting the pelvic floor muscles (PFMs) to enhance their responsiveness to a rise in intra-abdominal pressure. Pelvic floor muscle training (PFMT) is intended to strengthen and also target the coordination of the muscles of the sphincter complex [8]. Physical therapy has some documented benefits, but its disadvantages include a slow rate of improvement and poor compliance. Patients also need to be encouraged to perform Kegel exercises regularly, as they may not be correctly or consistently performed over time, which can reduce their effectiveness [9]. Remarkably, it has also been observed that over 30% of patients with SUI are unable to contract their PFMs on their first try [10].

An innovative strategy to solve UI symptoms is top flat magnetic stimulation (TOP FMS). This technology generates a magnetic field maintaining a homogeneous profile and prevents any regions with unequal stimulation intensity from producing strong, deep contractions in the muscles [11,12]. Moreover, it produces contraction of the PFMs by inducing electrical activity that depolarises the motor neurons and affects the blood circulatory system. The pelvic and/or pudendal nerves, and subsequently the external sphincters and/or the PFMs, are the primary stimulation targets in SUI. The benefits of this new technology include the avoidance of perceived pain, due to the low effect of magnetic stimulation on skin receptors and the fact that the patient does not need to undress because the magnetic field passes through clothing.

Another matter to be considered is that sacral nerves S2–S4 are the main sources of autonomic and somatic innervation for the urethra, vaginal wall, rectum, and PFMs. Therefore, an effective technique to regulate the pelvic floor and subsequently control the pelvic organs is employed to stimulate these roots [13]. The pelvic floor involves the confluence of three muscles: the puborectalis, pubococcygeus, and iliococcygeus muscles and the coccygeal muscle together with the levator muscle forms the pelvic floor [14]. The posterior midline of the hymen at the centre of the urethral meatus defines the urogenital hiatus. A digital examination is usually used to measure the urogenital hiatus, because increased genital hiatus size is linked to pelvic organ prolapse and levator anus muscle damage [15].

Weakness in the PFMs causes UI because they have an impact on urethral closure. This can be due to several reasons, such as hormonal fluctuations, type II myofibre atrophy, a decreased supply of proteins and calories, restricted physical activity, and modifications to protein synthesis, including myostatin [16].

Myostatin belongs to the TGF-β superfamily of transforming growth factors. TGF-β is well described as a cytokine that promotes the expression of type I collagen. Increased amounts of TGF-β following electrophysiological stimulation may therefore increase collagen expression [17,18]. However, it is unclear how electrophysiological stimulation caused the increase in TGF-β available locally in the urethra. Electrophysiological stimulation may enhance circulation, making TGF-β available locally. Wound healing can cause tissue degradation, releasing matrix-bound TGFs. This mechanism may contribute to TGF-β-induced SMAD (Sma-and-mothers-against-decaplegic) phosphorylation in UI. However, more research is needed to fully understand the molecular mechanisms of enhanced wound healing following electrophysiological stimulation [18]. Human skeletal muscle cells express myostatin, where it inhibits muscle development. Myostatin may have a negative effect by activating the PI3K/Akt signalling SMAD pathway [19]. For this reason, myostatin has been studied as a possible therapeutic target to prevent muscle mass loss in animal models and individuals affected by various muscle-wasting disorders because it induces muscular atrophy. Furthermore, collected data suggest that myostatin may be involved in a variety of physiological and pathological conditions, including obesity, insulin resistance, cardiovascular disease, chronic kidney disease, and the control of skeletal muscle development [20].

While the suppression of serum myostatin increases muscle growth and strength, myostatin concentration rises during skeletal muscle inactivity [21]. Moreover, the effects of ageing on skeletal muscle mass and function can be mitigated by therapeutic therapies that decrease myostatin signalling [20].

Myostatin can inhibit cell proliferation at the urethral sphincter level [22]. Prior research has shown that TOP FMS has a positive effect on the volume of the urethral rhabdosphincter [23]. Based on this scientific evidence, the aim of our research was to assess if TOP FMS would result in a change in serum myostatin levels. As secondary goals, we aimed at using trans-perineal three-dimensional (3D) ultrasound (US) to measure the length of the genital hiatus and any adjustments induced by TOP FMS contraction. Lastly, validated and dedicated questionnaires were conducted in order to define the degree of discomfort associated with a range of pelvic floor symptoms.

## 2. Materials and Methods

### 2.1. Study Design

#### 2.1.1. Study Population

In this clinical experimental prospective study, nineteen female patients with UI were enrolled from January to July 2023. The study was conducted at S. Marino Hospital, Republic of San Marino.

Inclusion criteria: Patients with SUI or UUI symptoms or various degrees of pelvic organ prolapse were considered eligible for the study.

Exclusion criteria: Patients with an implanted cardiac pacemaker, defibrillator, electronic/metal implants, neurostimulators, ferromagnetic prostheses, in a state of pregnancy, with recent deep venous thrombosis, weighing > 160 kg, acute inflammatory diseases, recent fractures in the area of treatment, fever, neoplasia, congestive heart failure, and arrhythmia were excluded. The patients’ UI status was first assessed by a gynaecologist. Following an interview that addressed pelvic floor dysfunction symptoms, family history of these symptoms, and/or surgical procedures, each participant was categorised as SUI or UUI based on dedicated questionnaires following the International Continence Society’s UI classification [24].

#### 2.1.2. Study Device

**Figure 1:**
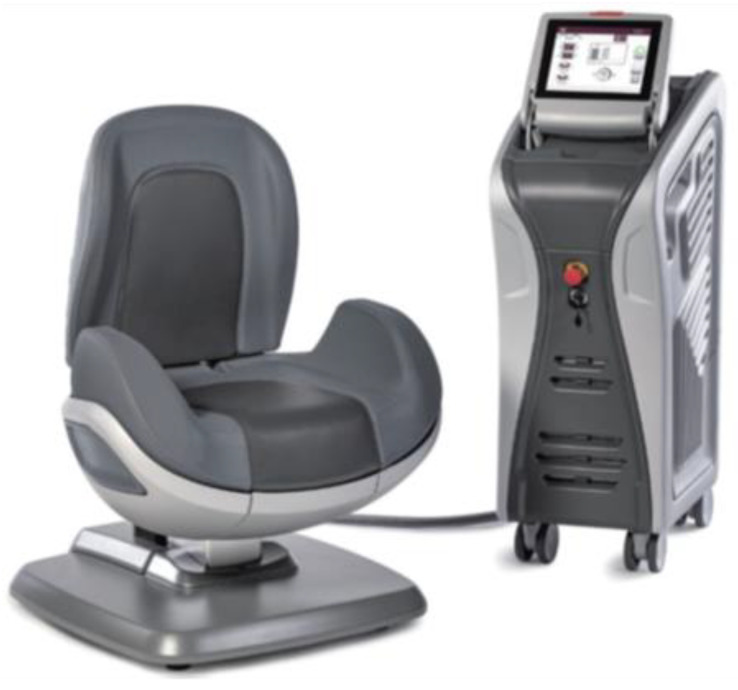
Representation of Dr. Arnold’s chair. Courtesy of DEKA M.e.l.a company, Calenzano, Italy.

Dr. Arnold (DEKA M.e.l.a, Florence, Italy) is a non-invasive device consisting of a main unit and a chair applicator that can be adjusted to provide deep stimulation of the PFMs (Figure 1).

To optimize the patient’s interaction with the electromagnetic stimulation and guarantee optimal comfort throughout the treatment session, the chair is made to allow the patient to adopt the proper therapeutic posture. The patient can remain clothed and should sit in the middle of the chair, erecting the spine (extension position), with legs flexed at the knees, thighs parallel to the floor, and feet flat on the floor (forming a 90° angle at the knee). She should also avoid wearing shoes. In this manner, the patient’s perineum is positioned at the centre of the seat, which facilitates the subject’s ability to feel the contraction of the PFMs and sphincter during electromagnetic stimulation [25]. Selectively stimulating the PFM TOP FMS technology is how the device operates. The exceptional uniformity of the magnetic field distribution over a larger region, which inhibits the formation of stimulation zones with varying intensities, allows for the recruitment of muscle fibres. Additionally, blood circulation benefits optimally from this kind of stimulation.

#### 2.1.3. Study Device Protocols and Quality Assessments

Two protocols were chosen for this investigation: Hypotonus/Weakness 1, in which the muscles strive to enhance trophism and tone, and Hypotonus/Weakness 2, which encourages the muscles to grow in volume and strength. Eight sessions, lasting thirty minutes each on a twice-a-week basis, were administered to each subject [23]. The first four sessions were carried out at an intensity level that allowed for the achievement of the ideal muscle contraction, following the manufacturer’s instructions. Patients were subsequently given more intense treatment if they tolerated it well and showed no signs of discomfort. The study staff performed a clinical re-evaluation on each patient after the eight sessions, corresponding to 1 month of follow-up (1 MFU).

The Pelvic Floor Bother Questionnaire (PFBQ) [26] (see Supplementary Materials) was employed to identify and rate the degree of discomfort associated with a range of pelvic floor symptoms. It is a nine-item questionnaire that covers the following conditions: dysuria, pelvic organ prolapse, faecal incontinence, faecal urgency and frequency, UI, SUI, and dyspareunia. Every response receives a score between 0 and 5, where higher numbers denote more serious bother. Every question has the same weight in the scoring system. Every response receives a score between 0 and 5, where higher numbers denote more serious bother. Every question has the same weight in the scoring system, and the overall score is between 0 and 45. The total score was converted by multiplying the mean score of the answered items by 20 to create a summary score that ranged from 0 to 100 (see Supplementary Materials). The PFBQ was administered before and at 1 MFU.

Lastly, before the session of treatment started, the patients were given information papers and validated consent.

#### 2.1.4. Myostatin Concentration

**Figure 2:**
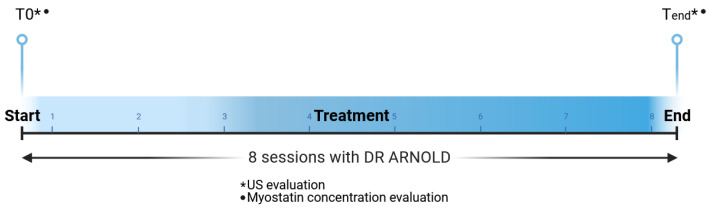
Study timeline. Every subject was treated with 8 sessions of electromagnetic stimulation. Blood samples were taken from patients before the treatment (T0) and at the end of the treatment cycle (Tend) (1 MFU) to measure the myostatin concentration. Moreover, ultrasound (US) tests at T0 and Tend were performed.

Blood samples were taken from patients before the treatment (T0) and at the end of the treatment cycle (Tend) (see Figure 2). Subsequently, the Myostatin ELISA protocol (RayBiotech Life, Peachtree Corners, GA, USA; cat. No: ELH-GDF8) was used for measuring myostatin in human serum and plasma. The collected samples were drawn into vacuum-sealed tubes containing EDTA anticoagulant, centrifuged for 15 min at 3000 rpm to extract plasma, and then frozen at −80 °C.

The results were then examined by the authors using microprocessor-controlled readers, designed to measure the light absorbance (optical density) of samples in 96-well microplates (Spectra and Rainbow Shell, Tecan, Cernusco Sul Naviglio, Italy). The sample colour intensity was measured at 450 nm.

A 96-well plate coated with an antibody specific to human myostatin is used in this assay. The immobilised antibody binds the myostatin in the sample to the wells when standards and samples are pipetted into them. After washing, the wells are incubated with biotinylated anti-human myostatin antibodies. HRP-conjugated streptavidin is added to the wells after the unbound biotinylated antibody has been washed away.

Following a second washing, the wells are filled with a peroxidase substrate tetramethylbenzidine solution (TMB) and colour changes occur in direct proportion to the amount of bound myostatin.

The colour is finally changed from blue to yellow by the acidic Stop Solution, and its intensity is measured at 450 nm. The increased photometric signal results from a high myostatin concentration.

#### 2.1.5. Quantitative Evaluation with Ultrasound

One clinician performed ultrasound (US) tests on the entire study group both before (T0) and after the eight treatment sessions (Tend), which corresponded to 1 MFU (see Figure 2). While the patient was in a supine position, a 3D transperineal–translabial ultrasound was performed. The hyperechogenic posterior surface of the pubic symphysis and the hyperechoic anterior border of the puborectalis muscle, which is located just posterior to the anorectal muscle, were used as markers. Using a 1–8 MHz 3D volumetric ultrasound transducer (CV1-8A), a Samsung HERA W9 and WS80 ultrasound (Samsung Healthcare, Seoul, Republic of Korea) was employed. For the acquisition angle, the maximum transducer angle of 120° was selected.

The patient’s legs were bent at the hips and knees, and the core transducer axis was positioned between the two labia majora at the level of the rear fork in the mid-sagittal plane.

PFM contraction induces reductions in anteroposterior (AP) diameter and hiatus area, and these modifications can be seen with transperineal ultrasound. The relaxation and contraction of the puborectalis muscle causes changes in the anorectal angle and hiatus size within the deep layer. In women with pelvic organ prolapse and incontinence, markers of PFM strength have been applied widely: bladder neck displacement, anorectal angle excursions, levator plate, and hiatus narrowing regarding the inferior border of the pubic symphysis. It has been found that there is a good correlation between the method used to assess pelvic organ descent during the Valsalva manoeuvre and clinical measurements of descent [27].

#### 2.1.6. Statistical Analysis

Student’s *t*-test and SPSS program version 25.0 (IBM Corp., New York, NY, USA) were used to analyse the collected quantitative data. Data are shown as means ± standard deviation (SD). A *p*-value < 0.01 was considered statistically significant with 99% confidence. The ES (Cohen’s d), calculated as the difference in the means of two groups divided by the weighted pooled standard deviations of the results (before vs. end of the treatment), was used for the comparisons [28]. Cohen-defined d measures of small, medium, and large ES were 0.2, 0.5, and 0.8, respectively [29].

## 3. Results

The patients enrolled in this study presented with 75% SUI and 25% UUI. The mean age of the patients was 49.4 ± 9.7 (range 27–72) and 32% of them were menopausal. In addition, 63.2% presented with pelvic organ prolapse of different grades [30] (uterine grade I, 13.3%; rectocele grade I, 6.7%; rectocele grade II, 13.3%; cystocele grade I, 33.3%; cystocele grade II, 33.3%) (see Table 1).

### 3.1. Myostatin Concentration

Data obtained from the Myostatin ELISA test shows that the myostatin level in blood serum significantly (*p* < 0.01) had decreased at the end of the treatment cycle (mean concentration: 14.43 ± 9.53 ng/mL at baseline vs. 7.18 ± 3.96 ng/mL after the last treatment session) (see Table 2). With 99% confidence, the myostatin mean was included in the interval (7.86, 21.01) at baseline. With 99% confidence, the myostatin mean was included in the interval (4.45, 9.91) at the end of the treatments (see Table 2). The calculation of Cohen’s d comparing patients before and at the end of the treatments suggested large effect sizes of this therapy. In fact, Cohen’s d for this study was more than 0.8 (0.99).

### 3.2. Quantitative Evaluation with Ultrasound

All study participants were monitored by US using 3D transperineal–translabial ultrasound for quantitative assessment of the study device’s efficacy before and after the treatment cycle (1 MFU).

Pre- and post-treatment improvements were compared using the distance between the hyperechogenic posterior surface of the pubic symphysis and the hyperechogenic medial–anterior border of the puborectalis muscle of the levator ani.

**Figure 3:**
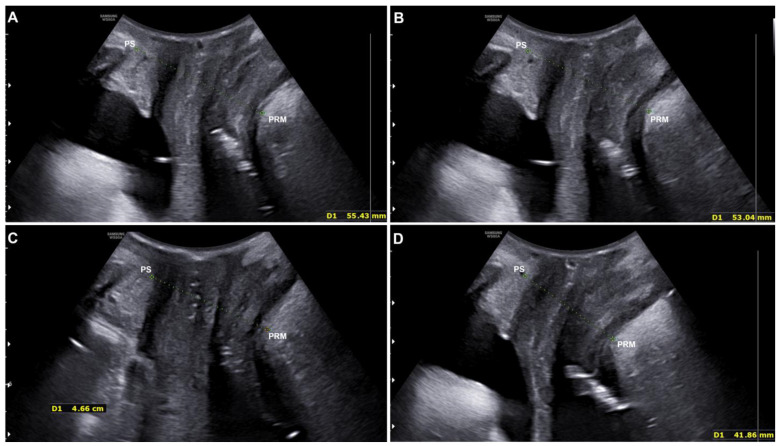
Ultrasound evaluation of a patient at rest before (**A**) and after (**B**) the treatment with electromagnetic stimulation. The ultrasound evaluation performed on the same patient under stress/contraction condition before (**C**) and after (**D**) the treatment with electromagnetic stimulation. The green lines represent the distance between the PS (pubic symphysis) and the PRM (puborectalis muscle).

The results showed a statistically significant (*p* < 0.001) distance reduction, both at rest (from 56.57 ± 6.52 mm to 54.32 ± 6.38 mm) and in a stress (contraction) condition (from 47.9 ± 4.86 mm to 44.80 ± 5.07 mm) (see Figure 3 and Table 3).

The distance between the inferior border of the pubic symphysis and the medial border of the levator ani (puborectalis muscle) was used to compare pre- and post-treatment improvements. PS = pubic symphysis; PRM = puborectalis muscle.

### 3.3. Qualitative Assessment: PFBQ

When comparing the pre-and post-treatment data, the PFBQ consistently showed a statistically significant (*p* < 0.01) decrease in the mean scores, indicating a steadily improving medical state. In particular, the mean score on this questionnaire (score range 0–45) was recorded at baseline as 38.51 (± 21.93), and it had decreased to 28.51 (± 13.87) immediately following the final treatment session (1 MFU) (see Table 4).

### 3.4. Side Effects

The study population did not report any side effects, including localised erythema or skin reddening, temporary tendon or joint pain, temporary muscular spasms, or skin pain.

## 4. Discussion

UI can be treated with a variety of non-invasive methods. One of these is the TOP FMS. Various studies have looked at the effectiveness and safety of this approach and how it affects patients’ quality of life. Several studies have shown very positive findings (reduced UUI, MUI, and SUI) using validated questionnaires and/or ultrasound tests [9,10,31,32,33]. This technology produces strong PFM contractions by depolarizing neurons and focusing electric currents on neuromuscular tissue. The S2–S4 roots of the sacral nerves provide the primary somatic and autonomic innervation of the PFMs, the rectum, the vaginal wall, the bladder, and the urethra. The spatial profile of the electromagnetic stimulation is a key feature that distinguishes Dr. Arnold from other devices. The symmetrical and homogeneous distribution of electromagnetic energy can reach the neuronal structures of the pelvic floor, such as the pudendal nerve (S2–S4), without superficially scattering.

Frigerio et al. (2022) underlined that patients who received TOP FMS had fewer incontinence episodes, improved urinary-related quality-of-life scores, increased urethral rhabdosphincter volume (enhancing the control of urine leakage) and more positive treatment outcomes than those who had performed Kegel exercises [33]. The primary muscle controlling the urinary tract is the urethral sphincter, which is made of a thick layer of striated muscle on the outside and a small layer of smooth muscle on the inside. The first underlying cause of SUI is thought to be the apoptosis of the exterior rhabdosphincter cells [34]. In addition, previous reports indicated that ageing decreased muscle strength through increased muscle fibre hypertrophy in humans [35].

Loss of apical vaginal support is positively correlated with and predicted by increasing genital hiatus size.

While an increase in the length of the levator ani hiatus may suggest damage to the puborectalis muscle, an increase in the length of the urogenital hiatus may suggest damage to the pubovisceral muscle [36]. It is probable that the inability of the levator ani muscles to close the urogenital hiatus, which is the source of pelvic organ prolapse, is a contributing factor to prolapse [37]. Our findings regarding the decrease in genital hiatus dimension, both in resting and contraction conditions, agree with several studies that have linked increased genital hiatus dimensions to the severity of prolapse in general, the risk of recurrence of prolapse surgery, and decreased levator anus muscle strength [38].

In this study, we have demonstrated how TOP FMS might reverse the effects of ageing-induced reductions in myocyte proliferation and myostatin expression. Myostatin is a member of the TGF-β protein family and negatively regulates myoblast proliferation and differentiation to myofibers. According to the literature, serum myostatin levels were 6.6818 ± 3.155 ng/mL in older females and 5.56 ± 2.9461 ng/mL in older males. A further published investigation found that adults with a mean age of 50.9 ± 14.0 years (similar to our study sample) had a serum myostatin level of 10.97 ± 6.77 ng/mL [39,40].

However, a literature analysis [41] indicates that a variety of potential physiological factors, including age, gender, and physical activity, may have an impact on blood myostatin levels. Genetic mutations that hider myostatin’s link to the latter’s receptors are therefore associated with muscle hypertrophy, playing a role in sphincter regeneration [42]. These outcomes may strengthen the muscles of the pelvic floor and urethral sphincter and aid in the recovery from functional declines such as SUI, which is partially due to muscular origins.

Yuan et al. (2020) have shown that myostatin can inhibit the proliferation and differentiation of muscle cells in vivo [43]. In addition, there is some evidence that myostatin concentration rises during skeletal muscle inactivity while serum myostatin inhibition leads to an increase in muscle mass and strength [21,44].

Furthermore, also in Yuan’s work, an increase in myogenesis was observed after suppressing the expression of myostatin, and consequently urethral continence and the thickness of the striated urethral muscle, as well as the ratio between muscles of the smooth and striated sphincter, had significantly improved.

Consistently with previous research [45], we noticed that serum myostatin concentration at the end of the treatment course was significantly (*p* < 0.01) lower compared to the baseline. In senior women with stress-related UI, effective results with extracorporeal magnetic innervation (ExMI) in a decrease in myostatin concentration and a reduction in the severity of UI were observed. These results indicate the possibility of using myostatin inhibition in clinical settings for UI. Furthermore, it has been demonstrated that myostatin inhibits the proliferation of human urethral rhabdosphincter satellite cells. It is therefore very plausible to hypothesize future medical treatments aimed at the transient and local reduction in myostatin to improve the proliferation and differentiation of satellite cells resident in the urethra [22].

After eight treatment sessions, the mean scores on the PFBQ had decreased, indicating a reduction in SUI and UUI symptoms based on the qualitative assessment and demonstrating good psychometric properties. This suggested that the patient’s quality of life would also benefit.

Compared to other UI treatment approaches, the TOP FMS offers several noteworthy advantages. Treatment methods like tension-free vaginal tape (TVT) or transobturator tape (TOT) have become more popular over the past 10 years, but they come with a number of potential drawbacks [46]. A recent published study [47] comparing TVT and TOT techniques indicated an overall complication rate of 3.51% in 16 cases and the complications were significantly more prevalent in the TVT group. Acute urinary retention occurred more frequently in the TVT group, with an incidence of 13.72%. Furthermore, their application is limited, as obesity has been identified as a significant predictor of failure.

Finally, both pharmacological and non-pharmacological modalities can be used in conjunction with the study technology [48]. Patients can remain clothed and seated in an ergonomic position due to the consistent release of the gradually supplied energy.

The study’s findings demonstrate that the treatment plan significantly improved the PFM tone and strength in patients with UI without causing any pain or adverse effects. The demonstration was conducted utilizing a validated questionnaire (PFBQ) for the qualitative analysis. Myostatin concentration and US exams were used for the quantitative analysis.

As a result, we can conclude that in current clinical practice, the Dr. Arnold device offers useful and efficient support for patients with various pathologies that could be widely used in the gynaecologic field.

### Study Limitations

This study has some limitations. Indeed, it would be interesting to extend the analysis to a broader cluster of patients, calculating their number using the prior sample size calculator and possibly compare it to a randomised control group. Moreover, quantitative evaluations should be performed after a few months’ follow-up to assess the variation in measurements compared with baseline data.

## 5. Conclusions

Flat magnetic stimulation reduces myostatin concentration and genital hiatus length, minimizing the severity of urinary incontinence. The results of the study show that without causing any discomfort or unfavourable side effects, the treatment plan significantly improved the PFM tone and strength in patients with UI.

## Figures and Tables

**Table 1:** Baseline characteristics of the study participants (*n* = 19). (UI = urinary incontinence; SUI = stress urinary incontinence; UUI = urge urinary incontinence; SD = standard deviation).

**Number of Patients**	19
**UI Type** **(%)**	SUI (75.00%) UUI (25.00%)
**Prolapse** **(%)**	63.15%
	Uterine (Grade II: 13.33%) Rectocele (Grade I: 6.66%, Grade II: 13.33%) Cystocele (Grade I: 33.33%, Grade II: 33.33%)
**Menopausal Patients** **(%)**	31.57%
**Average Age (Mean ± SD)**	49.42 ± 9.72 (range 27–72)
**Number of Pregnancies (Mean ± SD)**	1.31 ± 0.88 (range 0–3)

**Table 2:** Patients’ myostatin concentration levels measured with Myostatin ELISA protocol. Results before the treatment (T0) and at the end of the treatment cycle (Tend) are shown. (STDev = standard deviation).

	Myostatin Concentration		*p* Value
	T_0_ (ng/mL)	T_end_ (ng/mL)	
Mean	14.43	7.18	*p* < 0.01
STDev	9.53	3.96	*p* < 0.01

**Table 3:** Patients’ ultrasound (US) measurements acquired with the 3D transperineal-translabial ultrasound. Results before the treatment (T0) and at the end of the treatment cycle (Tend) are shown.

	At Rest (mm)	Interval Confidence Values	Contraction (mm)	Interval Confidence Values
Before treatment	56.57 ± 6.52	52.72; 60.42	47.49 ± 4.86	44.62; 50.36
End of the treatment	54.32 ± 6.38	50.55; 58.09	44.80 ± 5.07	41.81; 47.80
Significance (T0 vs. Tend)	*p* < 0.001		*p* < 0.001	

**Table 4:** The Pelvic Floor Bother Questionnaire (PFBQ) results before the treatment (T0) and at the end of the treatment cycle (Tend) are shown.

	Baseline	End of the Treatment	Significance
Questionnaire PFBQ	38.51 ± 21.93	28.51 ± 13.87	*p* < 0.01
Interval confidence values	25.20; 51.83	20.09; 36.94	

## Data Availability

Data that support the study findings are available on request from the corresponding author (I.F.).

## Supplementary Materials

The following supporting information can be downloaded at. https://www.mdpi.com/article/10.3390/medicina60091399/s1, The Pelvic Floor Bother Questionnaire (PFBQ).
