# Supplementary material for: Myostatin Changes in Females with UI after Magnetic Stimulation: A Quasi-Experimental Study
Source: Medicina (Kaunas). 2024 Aug 26;60(9):1399. doi: 10.3390/medicina60091399 (PMC11434281; doi:10.3390/medicina60091399)
Supplement: Supplementary file 1 [file medicina-60-01399-s001.zip › medicina-3054238-supplementary.pdf]

PELVIC FLOOR BOTHER QUESTIONNAIRE

Date: ___/___/_____

MR#:

Instructions: The following is a questionnaire about your pelvic health. All information is strictly confidential. Please mark (**X**) in the box that best describes your symptoms in the last month

1. Do you experience urine leakage (incontinence) related to physical activity, such as coughing, sneezing, laughing, lifting or changing position?
☐Yes ☐No
If yes, how much does it bother you?
☐ Not at all ☐ Only a little bit ☐ Somewhat ☐ A moderate amount ☐ A lot

2. Do you experience frequent urination (needing to urinate more often than usual; including the need to get up two or more times during the night because of a need to urinate)?
☐Yes ☐No
If yes, how much does it bother you?
☐ Not at all ☐ Only a little bit ☐ Somewhat ☐ A moderate amount ☐ A lot

3. Do you experience an abnormal strong feeling of urgency to urinate (sudden, compelling urge to void)?
☐Yes ☐No
If yes, how much does it bother you?
☐ Not at all ☐ Only a little bit ☐ Somewhat ☐ A moderate amount ☐ A lot

4. Do you experience urine leakage associated with the feeling of urgency (involuntary loss of urine occurring while suddenly having a strong urge to urinate)?
☐Yes ☐No
If yes, how much does it bother you?
☐ Not at all ☐ Only a little bit ☐ Somewhat ☐ A moderate amount ☐ A lot

5. Do you experience difficulty or discomfort in passing your urine?
☐Yes ☐No
If yes, how much does it bother you?
☐ Not at all ☐ Only a little bit ☐ Somewhat ☐ A moderate amount ☐ A lot

6. Do you experience the feeling of a bulge in the vagina (either the bladder, uterus, vagina or rectum)?
☐Yes ☐No
If yes, how much does it bother you?
☐ Not at all ☐ Only a little bit ☐ Somewhat ☐ A moderate amount ☐ A lot

7. Do you experience difficulty in completely emptying your bowels, such as needing to press near your vagina or rectum with your fingers to complete a bowel movement?
☐Yes ☐No
If yes, how much does it bother you?
☐ Not at all ☐ Only a little bit ☐ Somewhat ☐ A moderate amount ☐ A lot

8. Do you experience accidental leakage of fecal matter or gas?
☐Yes ☐No
If yes, how much does it bother you?
☐ Not at all ☐ Only a little bit ☐ Somewhat ☐ A moderate amount ☐ A lot

9. Are you sexually active?
☐Yes ☐No
If yes, does pain or discomfort curtail your ability to enjoy sex?
☐ Not at all ☐ Only a little bit ☐ Somewhat ☐ A moderate amount ☐ A lot
